# Multimodal management of meningioma: Experience from a low-middle-income country

**DOI:** 10.12669/pjms.41.13(PINS-NNOS).13371

**Published:** 2025-12

**Authors:** Syed Ahmad Faizan Bukhari, Aqeel Natt, Haseeb Mehmood Qadri, Zia Ul Rehman Najeeb, Momin Bashir, Dahir Ashfaq, Seeneen Bukhari, Tariq Imran Khokhar, Zubair Mustafa Khan, Sumira Kiran, Asif Bashir

**Affiliations:** 1 Syed Ahmad Faizan Bukhari, MBBS, FCPS. Punjab Institute of Neurosciences, Lahore, Pakistan; 2 Aqeel Natt, MBBS, FCPS. Punjab Institute of Neurosciences, Lahore, Pakistan; 3 Haseeb Mehmood Qadri, MBBS. Punjab Institute of Neurosciences, Lahore, Pakistan; 4 Zia Ul Rehman Najeeb, MBBS. Punjab Institute of Neurosciences, Lahore, Pakistan; 5 Momin Bashir, MBBS.Rutgers Robert Wood Johnson Medical School, New Brunswick, NJ, USA; 6 Dahir Ashfaq,Third Year Medical Student, Agha Khan University, Karachi, Pakistan; 7 Seeneen Bukhari, MBBS. Punjab Institute of Neurosciences, Lahore, Pakistan; 8 Tariq Imran Khokhar, MBBS, FCPS. Punjab Institute of Neurosciences, Lahore, Pakistan; 9 Zubair Mustafa Khan, MBBS, FCPS. Punjab Institute of Neurosciences, Lahore, Pakistan; 10 Sumira Kiran, MBBS, MS. Punjab Institute of Neurosciences, Lahore, Pakistan; 11 Asif Bashir, MBBS, MD, FAANS, FACS, (Diplomat American Board of Neurosurgery). Punjab Institute of Neurosciences, Lahore, Pakistan

**Keywords:** Brain Neoplasms, Developing countries, Meningioma, Pakistan, Resource-limited settings

## Abstract

**Objective:**

To evaluate the effects of multimodal management for intracranial supratentorial meningioma in terms of their surgical outcome and the functional status of patients in a low-middle-income country.

**Methodology:**

This retrospective, observational study analyzed consecutive cases of surgically excised supratentorial meningiomas at the Department of Neurosurgery, Unit-I, Punjab Institute of Neurosciences, from January 2022 to August 2024. Patients aged 18 or older with at least three months of follow-up were included, excluding those with incomplete records. Patient data, including clinical, radiological, surgical, and postoperative details, were collected via Google Forms and analyzed using SPSS for descriptive statistics.

**Results:**

This study included 60 patients with a mean age of 45.90±13.05 years, with the majority being males (65%,39). Gross total resection was achieved in 48.3%(29) of cases. Post-operative GCS scores were 13-15 in 95%(57) of patients, and 60%(36) experienced no complications. Histopathology showed 85%(51) of meningiomas were WHO grade I, with 70%(42) meningothelial and 10% (6) fibrous. Most (90%,54) of them did not require adjuvant therapy, with average pre-treatment and post-treatment KPS of 77.17±21.64 and 83.00±28.95, respectively. WHO grade II tumors (10%,6), including atypical and clear-cell types, were treated with adjuvant therapy in 5%(3) of all cases, with KPS scores of 76.32±19.54 and 82.13±26.35. WHO grade III tumors were found in 5% (3) of cases and were all treated with adjuvant therapy, with KPS scores of 75.13±19.34 and 80.21±26.72.

**Conclusion:**

Multimodal management of intracranial meningiomas based on comprehensive clinical assessment, timely neuroimaging workup, surgery, and appropriate referrals for adjuvant therapy based on WHO tumor grades reduces the risk of postoperative complications and improves the functional status of patients overall.

## INTRODUCTION

Meningiomas are the most common benign tumors of the central nervous system (CNS), arising from arachnoid cap cells.^1^ These tumors are the most prevalent non-malignant tumors of the CNS in adults, accounting for 38% of all brain tumors and 55% of non-malignant primary brain tumors.^2^ Meningiomas are slow-growing tumors that typically cause symptoms when they become excessively large or are located in specific brain sites, leading to pressure-related symptoms.^1^ In studies conducted in 2020 and 2023, it was proposed that meningiomas are most commonly found in the adult population, with an average age at diagnosis of 50.2 years, though they can also occur in the pediatric population.^1^,^3^ In a study conducted by He W et al., it was suggested that these tumors are more common in females, with a male-to-female ratio of 1:3.^3^

Treatment for meningiomas generally involves surgery, radiation, or a combination of both.^4^ Surgical removal is the most common treatment method, with gross total resection as the primary goal.^1^ However, resection can be challenging due to vascular compression and limited exposure to bridging vessels and cranial nerves.^1^ A study conducted by Wagner A et al. proposed that the location of these tumors can also affect the postoperative quality of life for patients.^4^ Meningiomas are the second most common tumors found in the cerebellopontine angle.^5^ In a study published in 2022, data was collected from 64 patients, with follow-up information available for 68.8% of them. Gross total resection was achieved in 30 patients (46.9%), and surgical complications occurred in 13 cases (20.3%). Post-operatively, 56 patients (87.5%) developed cranial nerve palsies, and tumor progression was observed in 10 patients (15.9%) after a mean follow-up of 102 months.^4^

There are a few studies from Pakistan documenting the outcomes of excision of meningioma, but none of them combines clinical aspects with radiology and histopathology with adjuvant options.^6^-^8^ The objective of the first study on multimodal management of supratentorial meningioma was to analyze the functional status of patients and postoperative complications in a low-middle-income country.

## METHODOLOGY

This is a retrospective, observational analysis of consecutively recruited cases of supratentorial meningioma (STM) who underwent excision at the Department of Neurosurgery, Unit-I, Punjab Institute of Neurosciences, Pakistan. This study is based on data collected from January 1, 2022, to August 30, 2024, using a non-probability consecutive sampling technique.

### Ethical approval:

It was received from the Institutional Review Board of Punjab institute of Neurosciences via Reference# 2071/IRB/PINS/Approval/2025, dated February 12, 2025.

### Inclusion criteria

- All patients with supratentorial meningioma, aged 18 years or above, irrespective of gender distribution, who underwent surgical excision of the lesion;
- Cases with at least a three-month follow-up.

### Exclusion criteria

- Patients with incomplete medical records;
- Lost to follow-up;
- Infratentorial and spinal meningioma.

### Operational definitions:

a) Multimodal management was defined to constitute relevant radiological investigations, surgical and adjuvant treatment, appropriate referrals and patient follow up.

b) Extent of resection was calculated using contrast-enhanced magnetic resonance imaging (CE-MRI) by measuring the tumor dimensions (length × width × height) and comparing preoperative with postoperative MRI findings. MRI was performed in all patients who underwent surgery, both preoperatively and immediately postoperatively. Gross total resection was defined as removal of >97% of the tumor volume on postoperative MRI. Near-total resection was defined as removal of >90% of the tumor volume on postoperative MRI. Subtotal resection was defined as removal of 70–89% of the tumor volume on postoperative MRI. Partial resection was defined as removal of <69% of the tumor volume on postoperative MRI.

c) Vascularity of the lesion was defined as mild when up to two cottonoids were used during surgery, moderate when three to five cottonoids were used, and high when more than five cottonoids were required. The standard cottonoid used in our institute measured 5 × 5 cm.

### Data analysis technique:

Data was collected from patients’ medical records and pictures archiving and communication system (PACS); it included demographics, radiological data, operative notes, clinical notes, post-operative stay in the hospital, post-op clinic follow-up, and rehabilitation/recovery. Furthermore, each patient was assigned a unique study ID, and data were collected using Google Forms (Google Inc., USA), with the patient ID used to ensure accurate data entry.

### Statistical analysis:

Statistical Package for Social Sciences (IBM SPSS Statistics for Windows, Version 24, USA) was used to analyze de-identified data. Categorical data were presented as frequencies and percentages. This included all the variables whose responses would be either “yes”/”no” or divided into categories. Continuous/quantitative data were presented as means with standard deviation (SD).

## RESULTS

Our study included 60 adult participants, with an average age of 45.90 years (SD = 13.05). Of the participants, 65%(39) were male, and 35%(21) were female. The most common symptom reported was headache, observed in 88.3%(53) of participants, followed by focal motor deficits (other than cranial nerves) in 65%(39), and seizures in 46.7%(28). The majority of patients (96.7%,58) had a pre-operative GCS score of 13-15, while 3.3%(2) had a pre-operative GCS score of 9-12. Regarding pre-operative neurological signs, 45%(27) of patients showed signs of intracranial hypertension (raised intracranial pressure signs such as headache, nausea, vomiting and sudden changes in mental status), 33.3%(20) experienced vision deterioration, and 25%(15) had impaired memory, reasoning, or behavior (Table-I).

**Table-I:** Descriptive analysis of the demographics and clinical presentation of patients with intracranial STM.

Variables	Percentage with Frequencies
Patient distribution	60
Mean age, years ± SD	45.90±13.05
Gender distribution, Male/Female, % (n)	65.0% (39), 35.0% (21)
Symptoms at Presentation, % (n)	Headache - 88.3% (53)
	Focal motor deficits other than cranial nerves - 65.0% (39)
	Seizures - 46.7% (28)
	Vomiting - 43.3% (26)
	Syncope - 6.67% (4)
	Loss of Consciousness - 10.0% (6)
	Focal cranial nerve deficits - 5.0% (3)
	Urinary Incontinence - 5.0% (3)
	Nausea - 1.67% (1)
	Blurring of Vision - 33.3% (20)
	Frontal Swelling - 1.67% (1)
	Diplopia - 1.67% (1)
***Pre-operative GCS, % (n)***	
3-8	0.0% (0)
9-12	3.33% (2)
13-15	96.7% (58)
Signs /Symptoms, % (n)	Signs of Intracranial Hypertension - 45.0% (27)
	Vision Deterioration - 33.3% (20)
	Impaired levels of memory,
	reasoning or behaviour - 25.0% (15)
	Proptosis - 6.67% (4)
	Focal cranial nerve deficits - 5.0% (3)
	Hemiparesis - 65%(39)
	Hearing loss - 1.67% (1)

Pre-operative neuroimaging analysis showed that 50 patients had a calcified lesion on CT scans. Nearly all patients (95%,57) had an isodense lesion on MRI T1, while 5% (3) had a hypodense lesion. All 60 patients showed a hyperintense lesion on MRI T2 and MRI FLAIR, with homogeneous enhancement on MRI with contrast, and no restricted diffusion on MRI DWI. The most common sites for the space-occupying lesions (SOLs) were the sphenoid ridge, affecting 26.7%(16) of patients, followed by the parasagittal region in 15% (9), and the convexity in 11.7%(7). Meningiomas were almost equally distributed between the right and left sides, with 43.3% (26) on the right, 40% (24) on the left, 13% (8) in the midline, and 1.7%(1) bilaterally. The average volume of the meningiomas was 138 cm³ (SD = 13.59) (Table-II).

**Table-II:** Descriptive analysis of pre-operative neuroimaging of patients with intracranial meningiomas.

Variables	Percentage with Frequencies
CT Findings, % (n)	83.3% had a calcified lesion
MRI T1 Findings, % (n)	95.0% (57) Isodense lesion, 5.0% (3) Hypodense lesion
MRI T2 Findings, % (n)	100% (60) had a hyperintense lesion
MRI Contrast Findings, % (n)	100% (60) had homogeneous enhancement
MRI DWI Findings, % (n)	100% (60) had no restricted diffusion
MRI FLAIR Findings, % (n)	100% (60) had hyperintense lesions, 15.0% (9) had minimal oedema
Site of SOL, % (n)	Sphenoid Ridge - 26.7% (16)
	Parasagittal - 15.0% (9)
	Convexity - 11.7% (7)
	Olfactory groove - 10.0% (6)
	Parafalcine - 10.0% (6)
	Falx - 8.3% (5)
	Petrous - 8.3% (5)
	Tentorial - 3.3% (2)
	Foramen Magnum - 1.7% (1)
	Orbital - 1.7% (1)
	Extracalvarial - 1.7% (1)
	Cavernous - 1.7% (1)
Side of SOL, % (n)	Right - 43.3% (26)
	Left - 40.0% (24)
	Midline - 13.3% (8)
	Bilateral - 1.7% (1)
Size of SOL, mean ± SD	138 cm^3^ ±13.59 cm^3^

DWI: diffusion-weighted imaging, FLAIR: Fluid-attenuated inversion recovery, SOL: space-occupying lesion.

Surgical excision was performed on all 60 patients. Almost half of the patients (48.3%, 29) underwent a gross-total resection, while 45% (27) had near-total resection, and 6.7% (4) had subtotal resection. Tumor consistency varied: 53.3% (32) of tumors were firm, 36.7% (22) were hard, and 10% (6) were soft. Tumor vascularity was categorized as mildly vascular in 30% (18), moderately vascular in 28.3% (17), and highly vascular in 41.7% (25). The gross total resection was achieved in the majority of cases, 48.3% (29), while Simpson Grade I resection was applicable in 93.3% (56) of patients. Post-operative GCS scores were 13-15 in 95% (57) of patients, and 9-12 in 5% (3). Regarding post-operative complications, 60% (36) of patients had no complications. Most patients (73.3%, 44) showed improvement post-surgery, 18.3% (11) remained static, and 8.3% (5) deteriorated. The deteriorated patients had impaired levels of memory, reasoning, or behavior. There were no mortalities (Table-III).

**Table-III:** Descriptive analysis of parameters of surgical management and post-operative sequelae of patients with intracranial meningiomas.

Variables	Percentage with Frequencies
Excisional Surgery, % (n)	100% (60)
***Consistency, % (n)***	10.0% (6)
Soft	53.3% (32)
Firm	36.7% (22)
Hard	
***Vascularity, % (n)***	30.0% (18)
Mild	28.3% (17)
Moderate	41.7% (25)
High	
***Extent of Resection, % (n)***	48.3% (29)
GTR	45.0% (27)
NTR	6.70% (4)
STR	0.0% (0)
PR	0.0% (0)
Biopsy	
***Post-operative GCS, % (n)***	0.0% (0)
3-8	5.0% (3)
9-12	95.0% (57)
13-15	
***Post-operative Complications, % (n)***	60.0% (36)
Nil	33.3% (20)
Persistent Neurologic Deficit	8.33% (5)
Impaired levels memory, reasoning or behavior	5.0% (3)
New-onset headache	3.33% (2)
Cerebral edema/Midline Shift	1.67% (1)
Surgical Site Infection	1.67% (1)
New-onset Signs of Intracranial Hypertension	1.67% (1)
Proptosis	0.0% (0)
CSF Leak	0.0% (0)
Hydrocephalus	
Surgical Outcome	Improvement - 73.3% (44)
	Static - 18.3% (11)
	Deteriorated - 8.30% (5)
	Deaths - 0.0% (0)

GTR: gross total excision, NTR: near total excision, STR: subtotal excision, PR: partial excision.

Histopathological results revealed that most meningiomas (85%, 51) were WHO grade I. This includes 70% (42) of tumors classified as meningothelial and 10% (6) as fibrous. Of these patients, 90% (46) were not referred for adjuvant therapy. The average pre-treatment KPS was 77.17 (SD = 21.64), and the post-treatment KPS averaged 83 (SD = 28.95). WHO grade II tumors were found in 10% (6) of cases, including 6.7% (4) atypical meningiomas and 3.3% (2) clear-cell meningiomas. These patients included 5% of the total cohort who were referred for adjuvant therapy. Their pre-treatment KPS was 76.32 (SD = 19.54), and the post-treatment KPS averaged 82.13 (SD = 26.35). Finally, 5% (3) of cases were classified as WHO grade III anaplastic meningiomas, all of which were referred for adjuvant treatment. These patients had a pre-treatment KPS of 75.13 (SD = 19.34) and a post-treatment KPS of 80.21 (SD = 26.72) .During follow-up, no recurrence was observed in these patients, (Table-IV).

**Table-IV:** Descriptive comparison of tissue pathology, adjuvant treatment, and Karnofsky Performance Status (KPS) Scale concerning grades of patients with intracranial meningiomas.

	Grade 1	Grade 2	Grade 3
WHO Tumor Grade, % (n)	85.0% (51)	10.0% (6)	5.0% (3)
Histopathology	Meningothelial meningiomas - 70% (42) Fibrous - 10% (6) Psammomatous - 5% (3)	Atypical - 6.67% (4) Clear cell - 3.33% (2)	Anaplastic - 5.0% (3)
Referral for Adjuvant Therapy	No - 90% (54)	Yes - 5% (3)	Yes - 5% (3)
Linear Accelerator (LINAC) Regimen	Nil	54 Gy in 30 fractions	59.4 Gy in 33 fractions
Pre-treatment KPS, mean ± SD	77.17 ± 21.64	76.32 ± 19.54	75.13 ± 19.34
Post-treatment KPS, mean ± SD	83.00 ± 28.95	82.13 ± 26.35	80.21 ± 26.72

KPS: Karnofsky Performance Status.

## DISCUSSION

This study represents a novel retrospective evaluation of meningioma patients managed with surgery and adjuvant therapies in Pakistan. To our knowledge, no prior Pakistani study has reported on multimodal meningioma management, as most local studies focus on clinical or pathological features alone.^6^,^7^

The mean age in our cohort was 45.9 ± 13.1 years, which is similar to that reported in several studies: 45.6 ± 8.2 years in Karachi, Pakistan, by Hamid et al., 43.7 ± 19.9 years across 32 centers in Pakistan by Shah et al., 46.8 ± 12.6 years in Bangladesh, and 47 ± 13.2 years in Nepal.^6^,^9^-^11^ Conversely, a large Chinese series reported a higher mean age of 51.4 years.^12^ In contrast to the typical female predominance seen in meningiomas, our series revealed an unusual slight male predominance (65%, 39 males), rather than the commonly reported 2:1 or higher female-to-male ratio in the literature.^6^,^7^,^10^-^13^ This diverges from regional LMIC reports in Pakistan (55% female), Bangladesh (70.6% female), and Nepal (68.7% female), as well as large international cohorts in China (70% female).^9^-^12^

Headache was the most frequent presenting symptom (88.3%, 53), paralleling rates of 91.2% in Bangladesh and 79.8% in Nepal, though lower values like 46.5% have been noted in smaller series.^6^,^10^,^11^ Seizures occurred in 46.7% (28) of our patients, higher than in Bangladesh (38.2%).^10^ The rarity of seizures in purely extracranial meningiomas underscores the larger lesion volumes seen in our cohort, likely reflecting delayed presentation.^10^,^11^ Signs of raised intracranial pressure, vomiting in 43.3% (26) and syncope in 6.7% (4), were also common, along with substantial visual deterioration (33.3%, 20) and proptosis (6.7%, 4). These aggressive presentations likely reflect delayed diagnosis due to socioeconomic constraints and limited neurosurgical access in Pakistan, a pattern similarly noted in other low- and middle-income countries.

Pre-operative imaging revealed that the lesions were predominantly supratentorial, with the sphenoid ridge (26.7%, 16 cases), parasagittal region (15.0%, nine cases), and convexity (11.7%) being the most commonly affected areas. This distribution aligns with the findings of Hamid et al. (convexity 32.6%, parasagittal 30.2%) and with Chinese data, which identifies convexity tumors as the most frequent (38.33%).^6^,^12^ All lesions showed homogeneous contrast enhancement and no diffusion restriction on MRI, while extensive peritumoral FLAIR edema in a subset of our patients suggests higher-grade biology, mirroring the high proportion (>83%) of tumors >5 cm reported in Nepal.^11^ Our mean tumor volume (138 cm³) exceeds volumes reported in anterior skull base cohorts (60 cm³) by Carvi et al.^14^ This late-stage imaging profile (large size with edema) again points to delayed referral and diagnosis in our patient population.

Surgical management aimed for maximal safe resection. We achieved gross total excision and near total excision in 48.3% (29) and 45.0% (27) of the cases respectively, compared to the 85.4% gross-total rate reported by Raman et al. in Nepal.^11^ This rate appears higher than in a prior study by Wadd et al. in Lahore, Pakistan, in which they reported only 17.5% Simpson I resections, with most tumors resected to Simpson II or III.^7^ The relatively high rates of extent of resection in our cohort may reflect both the predominance of WHO Grade I histology (85%, 51) and the use of modern microsurgical techniques. Notably, most of our tumors were WHO Grade I meningiomas with a high proportion of meningothelial subtype (70%, 42). This benign histology likely facilitated complete resection and may explain the low perioperative morbidity. Additionally, 36.7% (22) of lesions were hard during excision; however, all lesions were hyperintense on T2WI, suggesting that radiological signal intensity does not always correlate with intraoperative consistency.

Postoperative complications occurred in 40% (24) of patients, with persistent neurological deficits in 33.3% (20), comparable to the 55.9% complication rate in Bangladesh and 30% in Nepal.^10^,^11^ No deaths occurred in our cohort, a finding that compares favorably with the 1.7% mortality reported by Raman et al. in Nepalese patients and the 17.6% six-month mortality observed in a Bangladeshi series.^10^,^11^ Most patients maintained their level of consciousness and neurological function after surgery. For reference, 94.8% of patients in the Nepal series had a favorable outcome (mRS ≤3).^11^ Although direct score comparisons are limited by different metrics, our cohort’s outcomes were at least comparable if not better, likely due to the slightly younger age and aggressive management. Notably, 73.3% (44) of our patients improved postoperatively and only 8.3% (5) deteriorated, with zero perioperative mortality. These results underscore the benefit of aggressive, multidisciplinary care coordinated with perioperative management and rehabilitation.

Histopathologically, 85% (51) of tumors were WHO Grade-I, predominantly meningothelial (70%, 42) and fibrous (10%, 6), which closely parallels the high Grade-I prevalences reported in Bangladeshi (94.1%), Nepalese (98.7%), Kashmiri (87.8%), and Chinese (91.8%) cohorts.^10^-^13^ In contrast, one Pakistani study reports only 61.3% Grade-I meningiomas.^9^ The predominant histological subtype in our series was meningothelial (70%, 42), consistent with Mubeen et al., who also identified meningothelial meningiomas as the most common variant.^13^ Grade-II and III lesions constituted 10% (6) and 5% (3), respectively, and almost all higher-grade or residual tumors received adjuvant radiotherapy, Linear Accelerator (LINAC) regimen (5%, three cases in Grade-II; 5% and three cases in Grade-III) following the current guidelines.

Postoperative surveillance should begin with clinical and neuroimaging assessment at three months, followed by annual scans for five years then biennially in WHO Grade-I tumors, biannual scans for five years then annually in Grade-II, and imaging every three to six months in Grade-III, with discontinuation of follow-up if no radiographic progression is observed at five years.^15^ Reported five-year progression-free survival rates approximate 90% for Grade-I, 60–90% for Grade-II, and 28% for Grade-III tumors, whereas ten-year overall survival falls to 53% in grade II and effectively 0% in Grade-III despite optimal treatment.^16^ No perioperative mortality and 73.3% rate of postoperative improvement in our patients highlight a favorable short-term outlook, yet the typical delays to presentation in LMICs such as Pakistan may challenge long-term surveillance adherence. Prior literature further emphasizes that higher histological grade, larger tumor size, skull-base or frontal location, and seizure burden are key risk factors for both recurrence and diminished long-term health-related quality of life, which often manifests as persistent deficits in memory, attention, and executive function.^17^-^19^

### Strength and Limitations:

This study is the first Pakistani series analyzing meningioma management with both surgery and adjuvant therapy in a single cohort. The design is reproducible and includes detailed clinical, radiographic, and outcome data. Additionally, there were very few patients that were sent for adjuvant therapy. However, the retrospective design is subject to information bias, and the moderate sample size limits subgroup analyses and generalizability. We did not examine surgical approaches in detail, and follow-up was relatively short for detecting late recurrences.

## CONCLUSION

Multimodal management of intracranial meningiomas, including multidisciplinary surgery combined with tailored adjuvant therapy, yields excellent functional and oncological outcomes, paralleling global standards despite resource constraints. The younger age at presentation, higher volume, male predominance and variable tumor consistency despite homogenous radiological findings in Pakistani cohort are points of interest for future prospective studies.

### Author`s Contribution:

**SAFB, AN, HMQ, ZRN, MB, DA and SB:** Data acquisition and interpretation, drafted the manuscript.

**TIK, ZMK and SK:** Concept and design of the work, Critical review of the manuscript.

**AB:** Supervision, data analysis, critically reviewed the manuscript.

All authors have approved the final version to be published and agreement to be accountable for all aspects of the work in ensuring that questions related to the accuracy or integrity of any part of the work are appropriately investigated and resolved.
